# The Effect of Nitric Oxide Inhibition in Spinal Cord Injured Humans with and without Preserved Sympathetic Control of the Vasculature

**DOI:** 10.3389/fnins.2016.00095

**Published:** 2016-03-10

**Authors:** Rachael Brown, David Celermajer, Vaughan Macefield, Mikael Sander

**Affiliations:** ^1^School of Medicine, Western Sydney UniversitySydney, NSW, Australia; ^2^Neuroscience Research AustraliaSydney, NSW, Australia; ^3^Department of Medicine, Sydney Medical School, University of SydneySydney, NSW, Australia

**Keywords:** nitric oxide, spinal cord injury, nitro-L-arginine methyl ester, blood pressure, vascular conductance

## Abstract

Systemic pharmacological inhibition of nitric oxide (NO) causes a hypertensive response, which has been attributed both to inhibition of peripheral NO-mediated vasodilatation and to inhibition of central nervous NO-production leading to a later onset sympathetic vasoconstriction. In the present study we aimed to test the importance of these two mechanisms by comparing the time-courses of the hypertensive responses in spinal cord injured (SCI) subjects with varying degrees of loss of sympathetic vascular control depending on level of injury as well as able-bodied controls. We hypothesized that high level SCI with no sympathetic vasoconstrictor control would have an abbreviated time-course of the hypertensive response to the NO-inhibitor L-NAME, because they would lack the late onset sympathetic component to the hypertensive response. NO production was blocked in 12 subjects with SCI and 6 controls by intravenous infusion of L-NAME (1.55–2.7 mg/kg). We measured blood pressure, heart rate, and vascular conductance in the carotid, brachial, and femoral arteries before, during, and after 1 h of L-NAME in a 4-h protocol. Peak increases in mean arterial pressure were significantly larger in high level SCI vs. controls: 32 ± 6 vs. 12 ± 2 mmHg (both groups received 1.55 mg/kg). The decreases in vascular conductance in the brachial and femoral vascular beds were also larger in the high level SCI group, whereas decreases in heart rate and carotid conductance were not significantly different between the groups. There were no indications of any abbreviated responses in blood pressure or vascular conductance in the high level SCI compared to control. The mid level and low-level SCI subject had responses similar to controls. These data confirm previous reports that NO inhibition causes a larger increase in blood pressure in high level SCI, and extend these data by providing evidence for differences in vascular conductance in the limbs. The current data do not support an obligatory important role for sympathetic vasoconstriction in maintaining the hypertensive response to L-NAME in humans.

## Introduction

Resting blood pressure (BP) is brought about by a combination of cardiac output and peripheral resistance. The primary determinant of peripheral resistance is the internal diameter of the arterioles in the muscle and splanchnic vascular beds, which is controlled on a beat-to-beat basis by reflex vasoconstriction governed by the arterial baroreflex. Flow-mediated dilatation (FMD), which is largely mediated by nitric oxide (NO), is brought about by the shear stress of blood flow through resistance vessels and counteracts this vasoconstriction (Raitakari and Celermajer, 2000; Mullen et al., 2001; Patel and Celermajer, 2006; Kooijman et al., 2008). Of course, it is now known that the elusive endothelium-derived relaxing factor (EDRF) is in fact nitric oxide (NO), which acts directly on local smooth muscle and relaxes it (Palmer et al., 1987).

An increasing body of literature has demonstrated that NO is of functional importance for vasodilation and the normal control of BP in animals (Cunha et al., 1993; Sander et al., 1995, 1997), as well as in humans (Calver et al., 1992; Vallance et al., 1992; Sander et al., 1999; Young et al., 2009). Indeed, blocking nitric oxide synthase (NOS) by nitro-L-arginine methyl ester (L-NAME) in young normotensive individuals can cause arterial pressure to increase by on average 24 mmHg (Sander et al., 1999).

Previous studies in both animals and humans have indicated that NO inhibition causes an increase in blood pressure through inhibition of endothelium dependent vasodilation as well as central disinhibition of the sympathetic nervous vasoconstrictor tone (Cunha et al., 1993; Sander et al., 1995, 1997, 1999). The latter mechanism would then be caused by NO inhibition within the central nervous system, specifically in some of the brainstem centers involved in the central baroreflex signaling and in the brainstem centers involved in the central sympathetic outflow. While the peripheral mechanism is initiated as soon as NO inhibition begins, the central sympathetic activation caused by NO inhibition seems slower in onset, and may be delayed by 1–2 h after initiation of systemic NO inhibition (Sander et al., 1999). In a specific rat model of high spinal cord injury, previous studies have suggested that SCI rats have a lower hypertensive response to NO inhibition than controls (Sakuma et al., 1992; Togashi et al., 1992).

In the present study we aimed to test whether loss of sympathetic vasoconstrictor tone would change the magnitude or the time-course of the hypertensive response to NO inhibition in humans. We hypothesized that loss of sympathetic vasoconstriction in high-level spinal cord injured (SCI) patients would shorten the time-course of the hypertensive response to L-NAME.

Given that resting BP is often low in SCI, and more so in high-level SCI because of interruption of descending vasoconstrictor drive, one might have expected that NO production would be lower because of reduced perfusion pressure and shear stress through the resistance vessels. However, a recent study revealed that L-NAME caused a higher increase in blood pressure in high-level SCI individuals compared to controls (Wecht et al., 2007, 2008). This means that in high level SCI there is an increased NO production playing a greater role in resting blood pressure, either through increased basal NO production or due to the peripheral NO effects being unopposed by sympathetic vasoconstriction. Indeed, it has been shown that in patients with autonomic failure in whom there is no sympathetic output, blocking NO production also increases blood pressure more so than in healthy controls (Gamboa et al., 2008).

The present study was initiated in 2005 and we recruited patients and controls until 2008. At this point the above data from Wecht and colleagues was published, and the similar time-courses for the blood pressure responses in high level SCI and in controls they observed strongly suggested that there would be no discernible abbreviation of the hypertensive response to L-NAME in SCI patients with little or no sympathetic vasoconstrictor drive.

The current study confirms the conclusions reached by Wecht et al. (2007, 2008), but extends it in the following ways: (i) we compared the hemodynamic responses to L-NAME infusion in high-, mid-, and low-level SCI patients and able-bodied controls, and (ii) we used Doppler ultrasound to measure blood flow and conductance in brachial, femoral, and common carotid arteries in all groups.

## Methods

### Subjects

Studies were performed on 12 individuals with complete or incomplete (AIS B) SCI. One individual with high level SCI did not complete the protocol, due to headache in conjunction with a high blood pressure increase during L-NAME infusion. In this individual the L-NAME-infusion was stopped prior to end-point of the protocol and L-Arginine was given to revert the blood pressure raising effect of NO-inhibition. This individual was subsequently fine, but only a limited dataset from this trial was included in the analysis. We included a full dataset from the remaining 11 individuals with SCI. The SCI patients had suffered their injury 11 months-33 years previously and ranged in age from 22 to 63 years (mean ± SE − 42 ± 3 years). We recruited six healthy controls (26–63; 42 ± 5 years). All subjects were male with the exception of one SCI and one control subject.

We specifically wanted to address whether SCI lesion level (and thus the residual central sympathetic control) affected the magnitude and the time-course of the changes in BP, heart rate, and blood flow during blockade of NO production. Accordingly, data from groups of four high-level (C5-T2), four mid-level (T4-T6), and four low-level (T7-T10) SCI individuals were compared to the control group. These groups of SCI subjects, separated according to level of lesion, and the control subjects are shown in Table 1.

**Table 1:** **Subject characteristics and baseline vs. peak blood pressure and heart rate**.

**Subject characteristics**				**Baseline vs. peak response**					
				**Systolic**		**Diastolic**		**Heart rate**	
**Age**	**LEVEL**	**AIS**	**TSI**	**mmHg**				**bpm**	
38^*^	C5/C6	B	20y 2m	104	172	60	101	60	37
45	C5/C6	B	8y 8m	114	153	62	81	46	31
40	C5/C6	B	1y 2m	81	129	44	67	45	31
38	T2	A	5y	105	175	61	97	68	35
22	T4	A	11m	106	144	55	77	57	41
39	T4	A	6y	104	138	57	77	49	34
33	T4	A	8y 10m	113	139	57	77	63	41
37	T6	A	19y 10m	130	164	71	93	47	41
57	T7	A	32y 8m	141	161	78	95	48	39
32	T7	B	5y 3m	103	127	49	64	64	50
49	T8	A	6y 5m	135	171	82	103	72	51
63	T10	A	29y	126	153	72	89	53	43
32^*^	HC			105	115	62	76	76	56
44	HC			140	155	85	81	59	41
37	HC			117	131	64	85	56	48
26	HC			105	118	55	74	55	41
47	HC			135	155	84	94	58	46
63	HC			129	162	78	93	51	41

AIS, American Spinal Injury Association (ASIA) Impairment Scale; TSI, time since injury; HC, healthy control. The

* indicates the subject is a female.

Subjects with a history of cardiovascular disease, diabetes, hypertension, or other co-morbidity were excluded from the study. The SCI subjects continued their normal medication, none of which are known to interfere with the cardiovascular system. All participating subjects gave written (or oral and witnessed) informed consent, and the study received ethical approval from the Human Research Ethics Committees of the University of New South Wales and Prince of Wales Hospital.

### Procedure and analysis

All subjects were studied in the morning after an overnight fast, resting in a semi-reclined position with the room at a comfortable ambient temperature. The female SCI subject did remain in her motorized wheelchair for testing, but the wheelchair could be reclined so she was in the same position as the other subjects. All subjects emptied their bladder before the procedure, and those SCI subjects with an indwelling catheter were monitored throughout to ensure there were no drainage problems that could interfere with their BP.

Blood pressure was monitored non-invasively, using oscillometric arm cuff measurements, and radial artery tonometry (CBM-7000, Colin Corp., Japan). Heart rate (HR) was measured via standard Ag-AgCl ECG surface electrodes on the chest. Each BP and heart rate measurement was averaged over 5 min (typically 5 blood pressure measurements and heart rate averaged over 5 min). In the right common carotid, brachial, and femoral arteries ultrasound 2D-images of vascular diameter (in perpendicular long axis views, the calculated mean diameters were 2/3 diastolic diameter + 1/3 systolic diameter) and Doppler-derived mean blood velocities (automated mean blood velocity detection over 3–5 heart beats in steady state) were obtained with a 11 MHz linear-array vascular transducer (SonoSite Titan; Bothell, WA, USA). Flow was calculated as vascular cross-sectional area times mean blood velocity and conductance as flow divided by mean arterial pressure.

A 20 gauge intravenous cannula was inserted into the median cubital vein for infusion of (L-NAME) or L-arginine (a substrate for NO synthesis). After recording baseline measurements of BP, HR, and vascular conductances, L-NAME was administered over 60 min. After the early experience with a high blood pressure response in a patient with high-level SCI, the L-NAME dosing was adjusted such that patients with high-level SCI received 1.55 mg/kg, the remainder of the SCI patients (mid and low) received 2.7 mg/kg. Of the control subjects 4 were matched for gender and age with the high level SCI patients (see Table 1) and received 1.55 mg/kg and the last 2 controls received 2.7 mg/kg. This approach allowed for direct comparison of the responses in high-level SCI and controls with the comparison between all groups being performed by normalizing to the administered L-NAME dose (responses/mg of L-NAME/kg body weight). At the completion of the L-NAME-infusion, there were 120 min of additional recordings. After a total of 180 min (L-NAME infusion for 60 min and 120 min of follow-up) L-arginine, 200 mg/kg, was administered intravenously over 15–20 min.

In addition to baseline measures, BP, HR, flow, and conductance measurements were obtained at 30, 60, 90, 120, 150, 180, and 210 min. BP and HR were also measured at 15 and 45 min. The 210-min measurements represent the response to L-arginine. These time points were compared to baseline with regards to absolute values, and percentage changes from baseline were compared between groups. In the figures, values are presented as mean ± SE. For each parameter, we used a one-way, repeated-measures, ANOVA within the group, and a two-factor ANOVA with repeated measures on one factor (time) between the groups (Prism 6 for Mac, GraphPad Software Inc., USA). Differences were considered statistically significant at *p* < 0.05 with Dunn's correction for multiple comparisons.

## Results

### Hemodynamic effects of NO-inhibition with L-NAME in three patients with high-level SCI and four age and gender matched able-bodied controls

**Figure 1:**
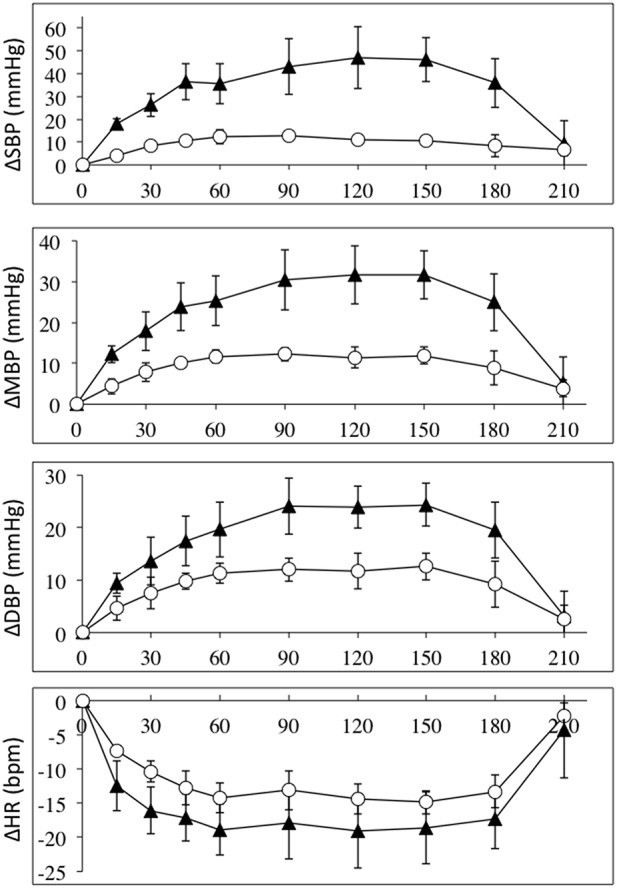
**Absolute changes (±SE) from baseline for systolic (top panel), mean (2nd panel), and diastolic (3rd panel) blood pressures (in mmHg); and heart rate (bottom panel) (BPM), during L-NAME, post L-NAME, and post L-arginine infusions in high-level SCI patients (black triangles) and matched controls (white circles)**. Time 0 is baseline measurements, the 15–60 min time points represent the L-NAME infusion, 90–180 min is post L-NAME infusion, and the 210 min time point represents post L-arginine infusion. For all variables L-NAME caused a significant change in both groups. There was a group effect for all blood pressure changes, such that the high-level SCI patients had larger increases in blood pressures compared to controls, whereas there was no group effect in the heart rate responses. There was insufficient power to test differences at specific time-points. We could find no indication of a differing time-course for the L-NAME effect in high-level SCI compared to controls.

Intravenous infusion of L-NAME (1.55 mg/kg) caused increases in arterial pressures, decreases in heart rate in all SCI and control subjects. Figure 1 depicts the absolute values for the changes in systolic, mean and diastolic blood pressures and heart rates in the group of high-level SCI patients and the matched controls. The increases in all pressures were 2–3 times higher in the SCI patients compared to the controls: the largest systolic pressure increase in SCI vs. controls: 47 ± 14 vs. 13 ± 1 mmHg [*F*_(1, 45)_ = 79.39; *p* < 0.0001]; the largest mean arterial pressure increase in SCI vs. controls: 32 ± 6 vs. 12 ± 2 mmHg [*F*_(1, 45)_ = 54.83; *p* < 0.0001]; the largest diastolic blood pressure increase in SCI vs. controls: 24 ± 6 vs. 12 ± 2 mmHg [*F*_(1, 45)_ = 26.65; *p* < 0.0001]. The differences in blood pressures at baseline, with the high-level SCI group starting much lower than the control group, were completely evened out after L-NAME. Despite the large differences in blood pressure responses, the heart rate changes were not significantly different between the groups: the largest decrease in SCI vs. controls was −19 ± 5 vs. −15 ± 2 bpm [*F*_(1, 45)_ = 8.707; *p* = 0.005]. When normalized to the increases in blood pressure, HR decreased by −0.8 and −1.6 bpm/mmHg in the high-level SCI and control groups, respectively; there was no significant difference in these falls in HR.

The average diameters of the common carotid, brachial, and femoral arteries (all three are conduit arteries) before and during L-NAME were not significantly changed in either group. There were some expected differences in absolute diameter between the groups, since the high level SCI group had substantially smaller femoral artery diameter (5.5 ± 0.4 mm) compared to the control group (8.2 ± 1.2 mm), whereas the brachial and carotid diameters were similar between the groups. The actual values before and during L-NAME for all groups (also the mid level and the low level SCI groups) are shown in Table 2.

**Table 2:** **Carotid, brachial, and femoral diameters at baseline and after the full L-NAME dose had been administered**.

	**Carotid diameter**		**Brachial diameter**		**Femoral diameter**	
	**Baseline**	**L-NAME**	**Baseline**	**L-NAME**	**Baseline**	**L-NAME**
High SCI	5.7±0.3	6.1±0.2	4.4±0.3	4.3±0.4	5.4±0.3	5.5±0.4
Mid SCI	6.3±0.04	6.2±0.1	4.4±0.3	4.4±0.3	5.8±0.3	5.8±0.3
Low SCI	6.6±0.4	6.5±0.5	5.0±0.2	4.9±0.2	6.2±0.2	6.2±0.2
Controls	6.1±0.2	6.1±0.2	4.3±0.3	4.3±0.3	9.0±0.8	9.0±0.8

The data for all four groups show that the diameters in these conductance vessels do not change when a NOS inhibitor is used to increase peripheral resistance.

**Figure 2:**
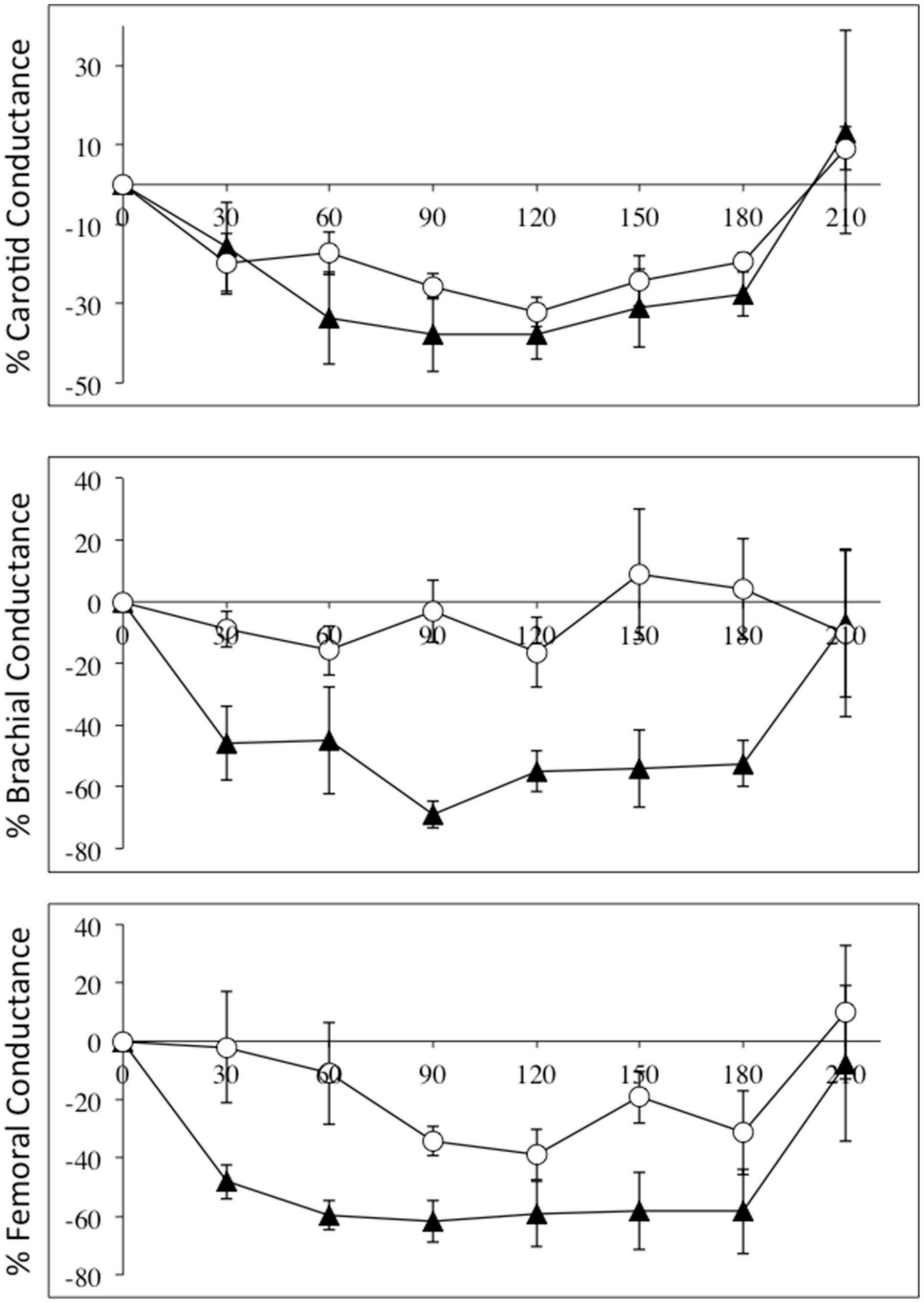
**Percentage changes (±SE) from baseline for carotid (top panel), brachial (middle panel), and femoral conductances, during L-NAME, post L-NAME, and post L-arginine infusions in high-level SCI patients (black triangles) and matched controls (white circles)**. Time 0 is baseline measurements, the 30–60 min time points represent the L-NAME infusion, 90–180 min is post L-NAME infusion, and the 210 min time point represents post L-arginine infusion. L-NAME caused large decreases in carotid, brachial, and femoral conductances in high-level SCI patients, whereas the L-NAME effects reached significance in the carotid conductance in controls. There were significantly higher decreases in conductance in the brachial and femoral conductances in the high-level SCI patients compared to matched controls.

The fact that diameter of these conduit arteries do not change during NO blockade means the changes in flow are related to changes in perfusion pressure and degree of downstream vasoconstriction. Vascular conductance measurements accounts for the changes in perfusion pressure in a linear fashion, and Figure 2 depicts the relative changes in conductance in the three vascular beds in the SCI and control groups.

Flow in the common carotid artery was not significantly changed in either group but carotid vascular conductance was decreased in both groups: change from baseline in SCI vs. controls: −38 ± 6 vs. −32 ± 4% [*F*_(1, 35)_ = 3.501; *p* = 0.0697]. Flow in the brachial artery was higher at baseline in high-level SCI compared to control, and the decrease was large and significant whereas there was no significant change in the control group: −35 ± 8 vs. −4 ± 15%. Brachial vascular conductance also changed markedly in high-level SCI but not significantly in controls: −55 ± 7 vs. −16 ± 12% [*F*_(1, 35)_ = 42.72; *p* < 0.0001]. In the femoral artery data showed decreases in blood flow: SCI vs. controls: −46 ± 6 vs. −24 ± 6%; and in vascular conductance: SCI vs. controls: −62 ± 7 vs. −34 ± 5% [*F*_(1, 35)_ = 22.90; *p* < 0.0001], a clear tendency for larger relative decreases in SCI compared to controls.

### Hemodynamic effects of NO-inhibition with L-NAME in high, mid, and low level SCI and able bodied controls

**Figure 3:**
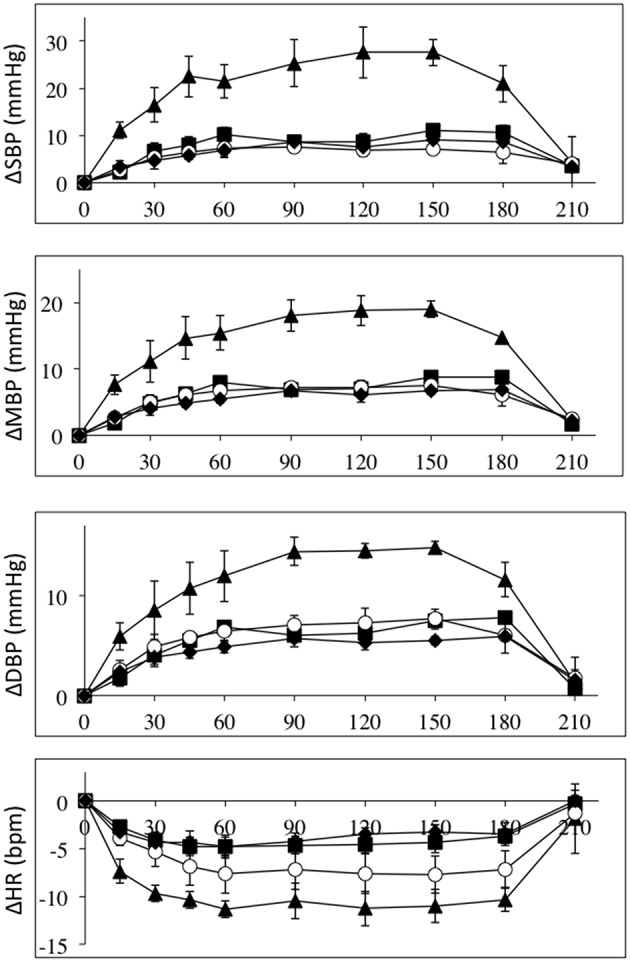
**All data are normalized to an L-NAME dose of 1 mg/kg**. Absolute changes from baseline (±SE) for systolic (top panel), mean (2nd panel), and diastolic (3rd panel) blood pressures (in mmHg); and heart rate (bottom panel) (BPM), during L-NAME, post L-NAME, and post L-arginine infusions in high-level SCI patients (black triangles), mid-level SCI patients (black squares), low-level SCI patients (black diamonds), and matched controls (white circles). Time 0 is baseline measurements, the 15–60 min time points represent the L-NAME infusion, 90–180 min is post L-NAME infusion, and the 210 min time point represents post L-arginine infusion. For all variables L-NAME caused a significant change in all groups. There was a group effect for all blood pressure changes, such that the high-level SCI patients had larger increases in blood pressures compared to the other three groups, whereas there was no group effect in the heart rate responses. There was insufficient power to test differences at specific time-points.

The effects of intravenous infusion of L-NAME (1.55–2.7 mg/kg) in the three patient groups and the control group were compared by normalizing to the L-NAME dose. Figure 3 depicts the absolute changes in systolic, mean and diastolic blood pressures and heart rates normalized to L-NAME dose in the four groups. These data clearly demonstrates that the high-level SCI group differs from the mid- and low-level SCI groups as well as controls. Roughly the blood pressure responses normalized to L-NAME dose are 2–3 times higher in the high-level SCI group compared to all three other groups. Heart rate decreases also tended to be larger in the high-level SCI group.

**Figure 4:**
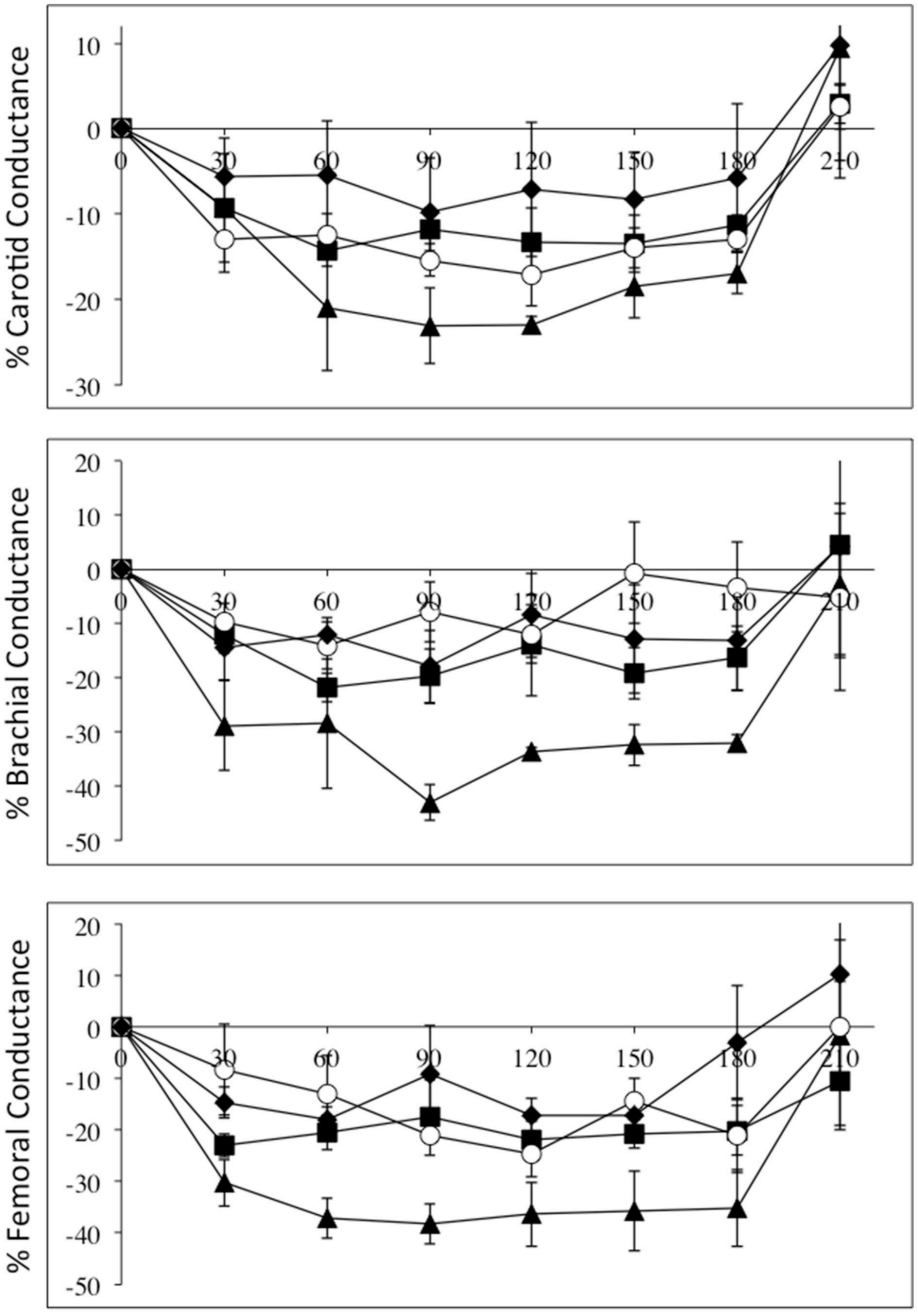
**All data are normalized to an L-NAME dose of 1 mg/kg**. Percentage changes (±SE) from baseline for carotid (top panel), brachial (middle panel), and femoral conductances, during L-NAME, post L-NAME, and post L-arginine infusions in high-level SCI patients (black triangles), mid-level SCI patients (black squares), low-level SCI patients (black diamonds), and matched controls (white circles). Time 0 is baseline measurements, the 30–60 min time points represent the L-NAME infusion, 90–180 min is post L-NAME infusion, and the 210 min time point represents post L-arginine infusion. L-NAME caused large decreases in carotid, brachial, and femoral conductances in high-level SCI patients and significant changes also occurred for the other three groups. There were no statistically significant differences between the groups.

Figure 4 depicts the relative changes in conductance in the three vascular beds, normalized to L-NAME dose, in all four groups. Again the high-level SCI subjects had larger decreases in brachial and femoral flow and conductance compared to controls. The mid-level SCI patients had very similar decreases in femoral conductance to the high level SCI patients, whereas the low-level SCI patients had decreases in femoral conductance that were similar to controls. However, there was no statistically significant difference between the changes in flow and conductances.

### L-arginine caused reversal of all hemodynamic effects of L-NAME in all individuals

As seen in Figures 1–4, there was reversal of the L-NAME effects in all groups with regards to all hemodynamic variables measured. This underscores the point that the L-NAME effects are most likely primarily—or exclusively—related to inhibition of NOS.

## Discussion

We have confirmed that systemic NO-inhibition in high-level SCI patients causes a significantly higher increase in blood pressure compared to matched control and compared to mid- and low-level SCI patients. Conversely, the bradycardic responses were not significantly different, suggesting the baroreflex-induced vagally-mediated bradycardia is intact in all groups. We have extended this finding by providing data that the relative decreases in vascular conductances in the brachial, and the femoral arteries are large in high level SCI. Although the decrease in femoral conductance in mid-level SCI is similar to high-level SCI, the blood pressure responses were different, suggesting that decreases in vascular conductance in the renal and splanchnic beds must be larger in high level SCI than in any of the other groups.

We set out to test the underlying mechanisms for the L-NAME induced increases in blood pressure by studying patients with SCI at different levels. In previous animal and human studies, evidence has been presented for a dominating peripheral mechanism where L-NAME blocks endothelially-derived vasodilatation, and a late-onset smaller effect of central sympathetic activation (Sander et al., 1997, 1999). The significance of such a late-onset sympathetic component has been unclear, although previous human studies suggested it could be responsible for as much as one third of the total response during the third hour after onset of NO-inhibition (Sander et al., 1999). The rationale in the present study was that, the higher the SCI level the smaller this late-onset sympathetically-mediated effect would be, since the sympathetic effector nerves are without central control below the level of the lesion (if the lesion is complete). During the latter part of our study period, Wecht et al. (2008) published data on the L-NAME induced hypertensive responses in SCI patients vs. controls. In their study, which showed a higher blood pressure increase in high level SCI patient, there were no discernible differences in the time-courses of the blood pressure responses between SCI and controls. Our data confirm this, as we found a higher increase in blood pressure in high-level SCI patients compared to controls and to mid- and low-level SCI patients. However, we found no suggestion of an abbreviated blood pressure response in high-level SCI, which would be expected if a late-onset central sympathetically mediated component was important. This underscores that NOs role in the control of vascular resistance is important, and that blocking this peripheral mechanism seems the more important factor in the hypertensive response to NO-inhibition.

What then is the explanation for the heightened blood pressure response in high-level SCI patients? A priori, we can think of two likely possibilities: (1) high-level SCI has a higher peripheral NO production leading to a higher NO-mediated vasodilation, which when blocked causes a higher increase in total peripheral resistance and hence blood pressure; (2) in subjects with an intact sympathetic nervous system, the L-NAME-induced increase in blood pressure leads to a baroreflex-mediated withdrawal of sympathetic vasoconstriction which buffers the increase in blood pressure, and in high-level SCI, this buffering is absent, because the sympathetic vasoconstrictor drive is absent due to the SCI.

We know that following SCI there is vascular remodeling, such that the luminal diameters of the carotid and femoral arteries are reduced (de Groot et al., 2006; Thijssen et al., 2008, 2012). Our data confirms that SCI patients have reduced diameter in the femoral arteries (Table 2). It seems, the explanation for this remodeling is primarily related to lack of muscle use, since patients with all levels of SCI (all in wheel chairs) have decreased femoral artery diameters, even though only high- and mid-level SCI have lack of sympathetic activation in the legs. In a previous study, Thijssen et al. (2008) have calculated that mean wall shear stress in a femoral conduit artery is about 30% higher in SCI compared to controls. If the decreased diameter of the conduit arteries is a good indicator for shear stress at the level of the resistance vessels and hence the shear stress induced NO production, it would be expected that all SCI groups had similar changes in femoral conductance. However, the high-level SCI had a larger L-NAME-induced decrease in femoral vascular conductance that the other two groups. Furthermore, the vascular remodeling does not knowingly occur in the renal arteries or the splanchnic vascular bed. These observations collectively speak against an increased peripheral NO production in high-level SCI compared to able-bodied controls. In a previous study, the intra-arterial infusion of the NO inhibitor L-monomethyl-L-arginine (L-NMMA) caused a similar decrease in flow and conductance in SCI and controls (Bleeker et al., 2005). Together this indicates that if differences exist for nitric oxide production in SCI and controls, the functional significance of such a difference seems limited.

Buffering the blood pressure raising effect of L-NAME by withdrawal of sympathetic vasoconstrictor tone seems logical. In this context, we would expect that in the resting human, an intact sympathetic innervation of the splanchnic and renal vascular bed would be paramount for such a buffering. Both the mid-level and the low-level SCI patients in the present study would be expected to have intact (or at least substantial) sympathetic innervation of the renal and splanchnic vascular beds. The similar blood pressure responses in mid- and low-level SCI and controls in the present study are consistent with this notion. Previous studies in humans have shown that muscle sympathetic activity is lowered during the initial phase of NO inhibition (Hansen et al., 1994; Lepori et al., 1999; Charkoudian et al., 2006). Recently, Wecht et al. (2008) reported decreases in noradrenaline levels (taken as an indirect measure of sympathetic withdrawal) in healthy subjects after L-NAME, and this buffering mechanism was not seen in high-level SCI patients that showed no significant changes in noradrenaline levels after L-NAME.

The limitations of the study are primarily related to the small number of subjects included in each group. A larger study was not feasible, because the L-NAME formerly produced for human use (by Clinalfa) was taken off the market around 2006. While we had some L-NAME in stock, we did utilize it during the course of this study, where subjects were enrolled up until 2008. We could have gained important knowledge if we had been able to obtain reliable data for mesenteric and renal vascular conductance, but although we aimed at this, the data quality was only sufficient in about 50%, and given the already small groups we elected not include any unclear data.

In conclusion, lack of central sympathetic control in high-level SCI does not cause changes in the time course of the hypertensive response to L-NAME. Furthermore, we confirmed that high-level SCI patients have a significantly more pronounced hypertensive response compared to the mid- and low-level SCI groups as well as controls.

## Author contributions

DC, MS, and VM designed the study. RB and MS performed the clinical study. RB, MS, and VM performed analysis of the collected data. RB, DC, and VM interpreted the data. RB and VM wrote the manuscript. All authors edited and approved the final manuscript.

### Conflict of interest statement

The authors declare that the research was conducted in the absence of any commercial or financial relationships that could be construed as a potential conflict of interest.
